# High-throughput sequencing reveals *Jatrorrhizine* inhibits colorectal cancer growth by ferroptosis-related genes

**DOI:** 10.1186/s12920-023-01619-3

**Published:** 2023-09-14

**Authors:** Lingyu Huang, Yu Sha, Wenken Liang, Chune Mo, Chunhong Li, Yecheng Deng, Weiwei Gong, Xianliang Hou, Minglin Ou

**Affiliations:** 1 Central Laboratory, Guangxi Health Commission Key Laboratory of Glucose and Lipid Metabolism Disorders, Guangxi Key Laboratory of Metabolic Reprogramming and Intelligent Medical Engineering for Chronic Diseases, The Second Affiliated Hospital of Guilin Medical University, Guilin, 541000 China; 2 Key Laboratory of Ecology of Rare and Endangered Species and Environmental Protection, College of Life Science, Ministry of Education of China, Guangxi Normal University, Guilin, 541000 China

**Keywords:** *Jatrorrhizine*, Colorectal cancer, High-throughput sequencing, Ferroptosis

## Abstract

**Background:**

Colorectal cancer is a malignant tumor that poses a serious threat to human health. The main objective of this study is to investigate the mechanism by which *Jatrorrhizine* (JAT), a root extract from *Stephania Epigaea Lo*, exerts its anticancer effects in colorectal cancer.

**Methods:**

We initially assessed the inhibitory properties of JAT on SW480 cells using MTT and cell scratch assays. Flow cytometry was employed to detect cell apoptosis. Differentially expressed genes were identified through high-throughput sequencing, and they were subjected to functional enrichment and signaling pathway analysis and PPI network construction. RT-qPCR was used to evaluate gene expression and identify critical differentially expressed genes. Finally, the function and role of differentially expressed genes produced by JAT-treated SW480 cells in colorectal cancer will be further analyzed using the TCGA database.

**Results:**

Our study demonstrated that JAT exhibits inhibitory effects on SW480 cells at concentrations of 12.5µM, 25µM, 50µM, and 75µM without inducing cell apoptosis. Through high-throughput sequencing, we identified 244 differentially expressed genes. KEGG and GO analysis of high-throughput sequencing results showed that differentially expressed genes were significantly enriched in MAPK, Wnt, and P53 signaling pathways. Notably, JAT significantly altered the expression of genes associated with ferroptosis. Subsequent RT-qPCR showed that the expression of ferroptosis genes SLC2A3 and ASNS was significantly lower in JAT-treated SW480 cells than in the control group. Analysis by TCGA data also showed that ferroptosis genes SLC2A3 and ASNS were significantly highly expressed in COAD. The prognosis of SLC2A3 was significantly worse in COAD compared to the normal group. SLC2A3 may be a core target of JAT for the treatment of COAD.

**Conclusions:**

JAT can inhibit COAD growth by ferroptosis-related genes. And it is a potential natural substance for the treatment of COAD.

**Supplementary Information:**

The online version contains supplementary material available at 10.1186/s12920-023-01619-3.

## Background

Colorectal cancer (COAD) is a prevalent and highly malignant tumor affecting humans, with increasing mortality and morbidity rates [1]. Current treatments options for COAD primarily involve Surgery, postoperative radiotherapy, and chemotherapy. However, despite these interventions, patients often experience tumor recurrence and face poorer survival outcomes [2, 3]. Due to the limited clinical efficacy and various side effects associated with existing therapeutics strategies, there is an urgent need to develop novel drugs for COAD patients.

*Stephania Epigaea Lo*, is a plant belonging to the Menispermaceae of Stephania, is renowned as a traditional Chinese herb. It is primarily found in Guangxi and Yunnan Province in China. The tuberous root of this plant has garnered significant attention for its medicinal properties. *Jatrorrhizine* (JAT), an anti-cancer tetrahydroisoquinoline alkaloid, is extracted from the *Stephania Epigaea Lo* [4, 5]. Additionally, previous research has indicated that JAT exhibits therapeutic effects in treating vascular inflammation [6], ulcerative colitis [7], anti-diabetic [8], and anti-obesity effects [9]. Importantly, JAT has demonstrated low systemic toxicity in vivo [5].

According to the literature, ferroptosis is a distinctive mode of cell death characterized by lipid peroxidation. It stands apart from apoptosis, necrosis and autophagy [10, 11]. Furthermore, emerging evidence suggests a significant association between ferroptosis and cancer development, invasion, and metastasis [12]. Consequently, targeting ferroptosis has gained considerable attention as a potential avenue for innovative cancer treatments [13]. Recent studies have specifically indicated that inducing ferroptosis holds promise in inhibiting the growth and progression of COAD [14–16]. As such, we firmly believe directing therapeutic interventions towards ferroptosis represents a compelling and prospective strategy for managing COAD.

Traditional Chinese Medicine (TCM) has gained increasing significance in oncology drug therapy research. However, the specific mechanism underlying the anticancer effects of JAT remains unclear. In our study, we sought to elucidate the inhibitory effect of JAT on colorectal cancer through a comprehensive approach. Initially, we employed MTT, scratch assay, and apoptosis assay to assess JAT’s inhibitory potential in colorectal cancer. Subsequently, we employed high-throughput sequencing to analyze the differentially expressed genes in SW480 cells treated with JAT. Through bioinformatics analysis, we validated the mechanism of action involving ferroptosis-related genes in COAD. Our aim is to synergize TCM treatment with high-throughput sequencing to unravel the regulatory mechanism underlying the therapeutic properties of JAT in COAD.

## Materials and methods

### Materials

**Fig. 1:**
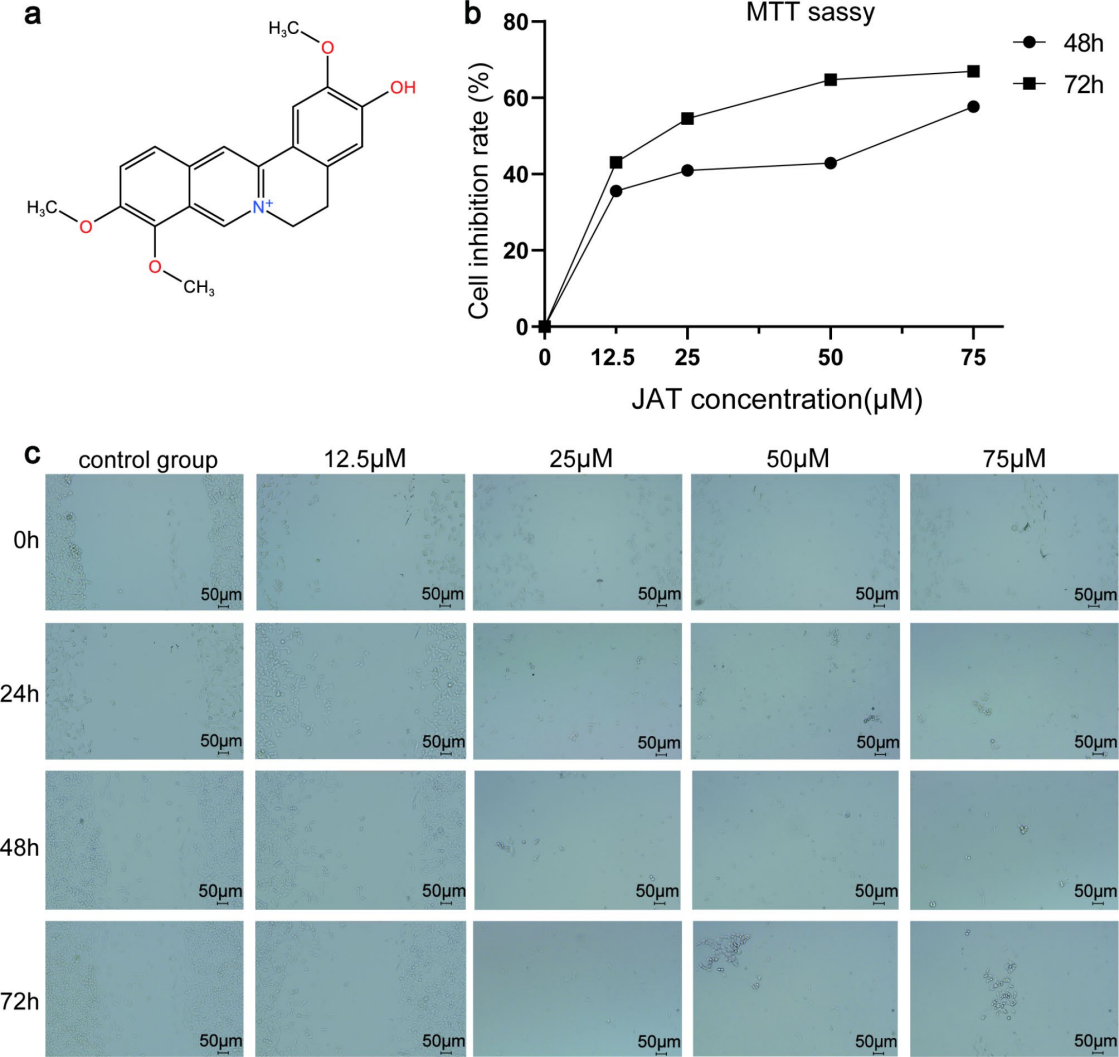
JAT intervention in SW480 cells experiments. (**a**) JAT structural formula. (**b**) MTT assay. (**c**) Cell scratch assay. Imaging at a magnification of 20× with a scale bar of 50 μm

JAT has a chemical formula of C_20_H_20_NO_4_^+^ and was purchased from Chengdu Desite Biotechnology (Chengdu, China). It has a CAS number 3621-38-3 and a purity of 99.82%. The structural formula of JAT is depicted in Fig. 1a. The SW480 human colon cancer cell line was sourced from our laboratory’s storage.

### MTT assay to determine the concentration of JAT that inhibits the proliferation of SW480 cells

The SW480 cells were diluted to a concentration of 5 × 10^4^ cells/mL and transferred to a 96-well plate. Afterward, the cells were treated with varying concentrations of JAT including 12.5 µM, 25 µM, 50 µM, and 75 µM for two and three days. To assess cell viability, 0.5 mg/mL MTT agent was added to the cells and incubated for 4 h. Subsequently, the cells were detached using DMSO, and the absorbance was measured at 490 nm using an enzyme-labeled instrument. The cell inhibition rate was then calculated based on the obtained measurements.

### Assessment of the invasive and migratory effects of JAT on SW480 cells by cell scratching assay

SW480 cells were seeded at a density of 6 × 10^5^ cells/mL in a 6-well plate and incubated for 24 h to allow for cell attachment and growth. After reaching confluence, a scratch was created in the SW480 cells layer using a sterile 200 µL pipette tip. Subsequently, the SW480 cells were treated with JAT at concentrations of 12.5 µM, 25 µM, 50 µM and 75 µM for a duration of three days. The same locations of the scratch were imaged again 24, 48, and 72 h after scratch initiation to monitor the progress of scratch repair.

### Flow cytometry assay to study the apoptosis effect of JAT on SW480 cells

A total of 6 × 10^5^ cells were seeded per well in a 6-well plate and treated with JAT at the specified concentrations for a duration of three days. Following the treatment, the cells were collected, harvested, and washed once with cold PBS. Subsequently, the cells were stained with Annexin V-FITC/PI to assess cell apoptosis rate. Flow cytometry was utilized to detect and analyze the apoptosis levels of the cells.

### High-throughput sequencing and identifying ferroptosis-related genes

High-throughput sequencing was conducted on SW480 cells that had been treated with JAT for a duration of three days. Total RNA was extracted and subsequently sequenced by BGI-ShenZhen. Differentially expressed genes were identified based on the following criteria: |log2FC|>=1 and FDR < = 0.001. Enrichment analysis, including KEGG and GO analysis, as well as the construction of PPI network, was performed on these differentially expressed genes.

Furthermore, a set of ferroptosis-related genes were obtained from various databases such as FerrDb, NCBI, Genecards and KEGG databases, and considered as candidate genes. The mRNA data and clinical information of 514 patients were acquired from the TCGA database. Data were analyzed utilizing R software to identify differential ferroptosis-related genes in JAT-treated SW480 cells.

### RT-qPCR assessment of ferroptosis-related gene expression in JAT-treated SW480 cells

After treating the SW480 cells with JAT. RNA extraction was carried out, and the extracted RNA was stored at -80℃. Subsequently, the mRNA was reverse transcribed into cDNA, which was further used for RT-qPCR experiments. The RT-qPCR analysis was conducted using the ABI7500 Real-time PCR instrument. To ensure accurate normalization, GAPDH was used as the internal control. The relative gene expression levels were determined using the 2^−∆∆Ct^ method. Graphs presenting the results were generated using GraphPad Prism 8 software. The primer sequences utilized in the RT-qPCR experiments can be found in Table S1.

### Analyses of ferroptosis-related genes expression in COAD and prediction of patient survival prognosis using the TCGA dataset

To analyze the differential expression between tumor and normal tissues, we utilized the “limma” and “beeswarm” R packages. Additionally, the “survivor” R package was employed to perform gene survival analysis in COAD. Furthermore, we investigated the expression of genes across different clinical stages. And the results were visualized using an appropriate R package. In addition, we constructed prognostic models using ferroptosis-related genes to predict the prognosis of COAD patients. The TCGA cohort was randomly divided into two groups, namely the high-risk group and low-risk group, using R packages. Survival analysis was performed using the “ survival” R package to assess the association between risk scores and patient outcomes. ROC curves were generated at 1, 5, and 10 years to evaluate the difference in survival between the patients over time. Additionally, risk curves were employed to explore the potential correlation with patient survival time. Furthermore, the relationship between differentially expressed genes for ferroptosis and survival prognosis of patients was analyzed using univariate Cox and multivariate Cox. Statistically significant was defined as *p*-value < 0.05 for the above-mentioned result. This comprehensive analysis allowed us to gain insights into the gene expression patterns, differential analysis, survival prognosis, and clinical stage-associated expression in COAD.

## Results

### JAT inhibits cell proliferation in SW480 cells

The MTT assay is a widely used method for assessing cell viability. It is a quantitative colorimetric assay that indirectly measures the survival and proliferation of cells by detecting viable cells [17]. In our study, we utilized the MTT assay to investigate the effect of JAT on the growth of colorectal cancer cells. SW480 cells were treated with different concentrations of JAT and co-cultured for specific durations. Our findings revealed that JAT exhibited inhibitory properties on SW480 cells after 48 and 72 h for intervention. Moreover, we observed that the inhibitory effect was both time- and dose-dependent. Higher concentration of JAT and longer intervention time resulted in more pronounced inhibition of SW480 cells. The results are depicted in Fig. 1b, providing visual representation of the observed inhibitory effects of JAT on SW480 cell growth.

### JAT inhibition of metastasis and invasion of SW480 cells

Cancer cell migration and invasion play pivotal roles in the progression of cancer. To investigate the impact of JAT on the migration ability of colorectal cancer cells, we performed a cell scratching assay. The results, depicted in Fig. 1c, demonstrated a significant reduction in the migration ability of SW480 cells upon JAT intervention compared to the control group. It is noteworthy that the cells in the co-culture of SW480 cells with 25 µM JAT for 24 h resulted in a remarkable decrease in cell viability.

### Effect of JAT on the apoptosis of SW480 cells

**Fig. 2:**
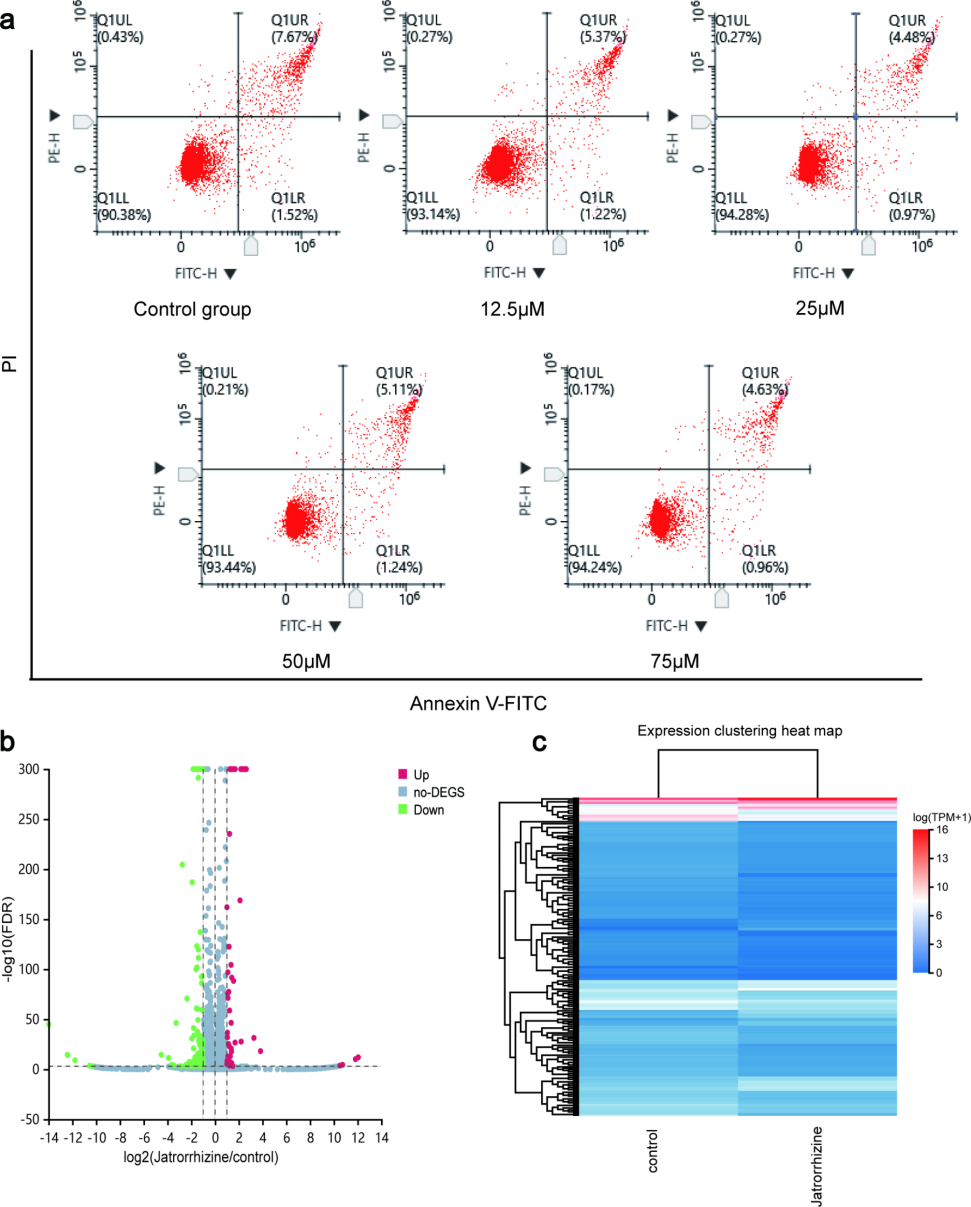
Detection of cell apoptosis and visualization of sequenced genes. (**a**) The effect of JAT on cell apoptosis of SW480 by flow cytometry. Distribution of high-throughput sequenced genes in volcano (**b**) and heat map (**c**)

After treating SW480 cells with different concentrations of JAT (0, 12.5, 25, 50, and 75 µM) for 72 h, flow cytometry was performed, and the results were presented in Fig. 2a. The cell apoptosis rates of the control group, 12.5 µM JAT group, 25 µM JAT group, 50 µM JAT group, and 75 µM JAT group were found to be 9.19%, 6.59%, 5.45%, 6.35%, and 5.59%, respectively. Importantly, the results indicated that JAT treatment did not significantly promote apoptosis compared to the control group.

### High-throughput sequencing data analysis

**Fig. 3:**
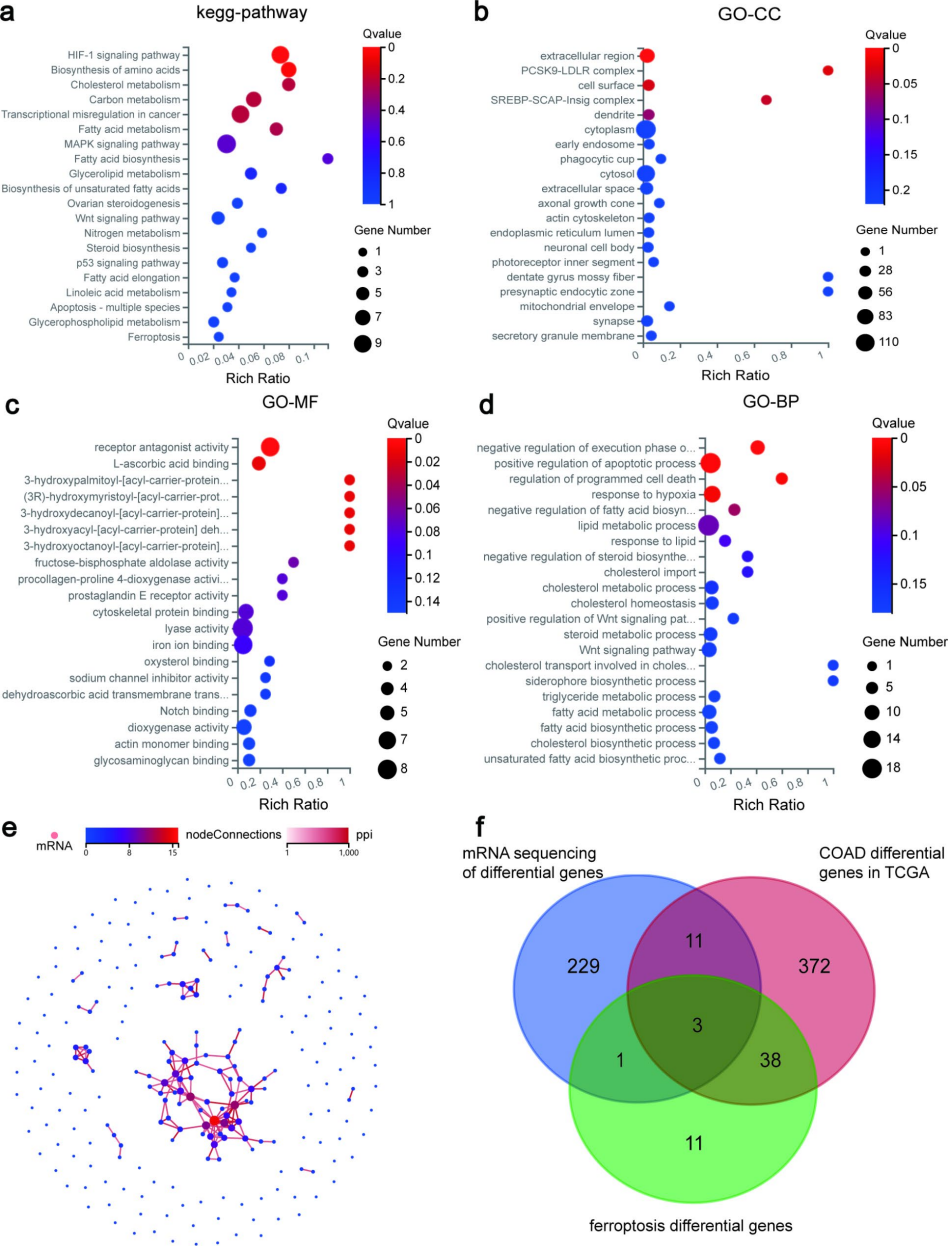
Identification and analysis of differentially expressed genes by high-throughput sequencing. (**a**) KEGG enrichment analysis. GO enrichment analysis including CC (**b**), MF (**c**), and BP (**d**). (**e**) PPI network of differentially expressed genes. (**f**) Venn diagram, screening for critical ferroptosis genes

The high-throughput sequencing data analysis results were visualized through volcano plots (Fig. 2b) and clustered heat maps (Fig. 2c). In the Dr.TOM system, we identified 244 differentially expressed genes (Table S2). KEGG enrichment analysis revealed that differentially expressed genes were mainly associated with lipid metabolism, including pathways such as Cholesterol metabolism, Fatty acid metabolism, Fatty acid biosynthesis, and Glycerophospholipid metabolism, Glycerolipid metabolism (Table S3). To present the results, we select 20 of these pathways and created bubble diagrams (Fig. 3a). Notably, differentially expressed genes were also enriched in ferroptosis and ferroptosis-related pathway MAPK signaling pathway. In addition, there are Wnt and P53 signaling pathway. GO analysis also indicated an association with iron ion binding (Fig. 3b-d). Based on these findings, we hypothesize that the inhibitory effect of JAT on SW480 cells proliferation may be related to ferroptosis. Furthermore, the PPI network of the differentially expressed genes was constructed and visualized in Fig. 3e, where the nodes represent proteins and the connecting lines indicate the interactions between these proteins. The PPI network highlights the interconnectedness among most of the proteins.

### Identification of SLC2A3 and ASNS as critical ferroptosis genes in the inhibition of SW480 cells proliferation by JAT

**Fig. 4:**
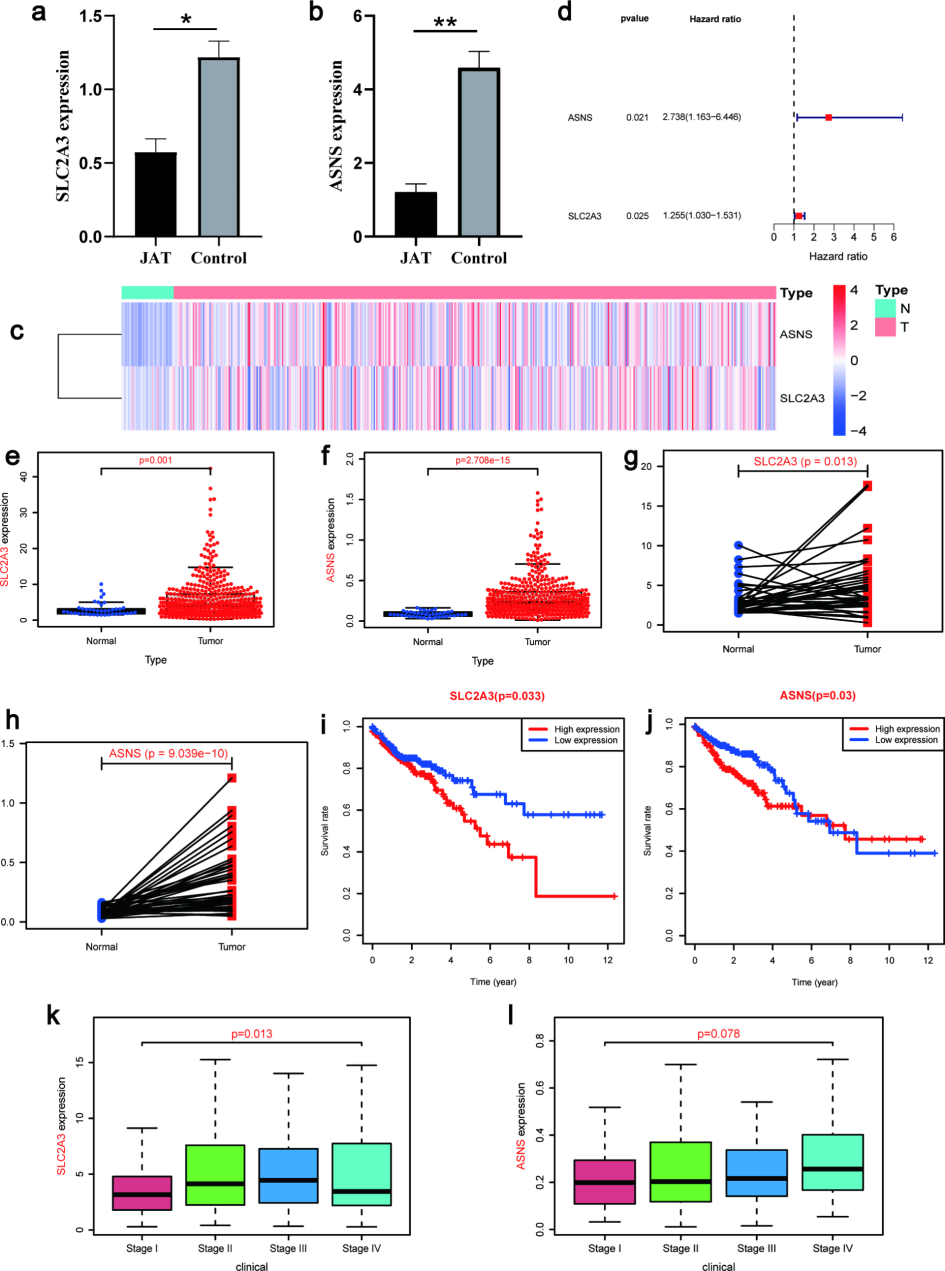
Identification of genes associated with differential ferroptosis. The RT-qPCR experiments for SLC2A3 (**a**) and ASNS (**b**). (**c**) Heat map of critical ferroptosis gene expression in the COAD tumor group and normal group. (**d**) Forest diagram of critical ferroptosis genes. The expression of SLC2A3 (**e**) and ASNS (**f**) in COAD was studied using the R package. (**g**-**h**) Paired difference analysis. (**i**-**j**) Survival analysis. (**k**-**l**) Clinical staging analysis. *, P < 0.05; **, P < 0.01; ***, P < 0.001; P < 0.05 was considered statistically significant

From the TCGA dataset, we identified 424 differential expressed genes in COAD and 53 ferroptosis-related prognostic genes (Table S4 and S5). By intersecting these three gene sets, we obtained a list of potential prognostic genes. Among them, three genes, namely SLC2A3, DDIT3, and ASNS, were identified (Fig. 3f). The RT-qPCR results showed that SLC2A3 and ASNS were lowly expressed after JAT-intervened SW480 cells compared to the control group (Fig. 4a-b). It is important to note that both were statistically significant. Heat maps and forest plots were generated to visualize the expression levels and statistical significance of SLC2A3 and ASNS, revealing that they were significantly upregulated in COAD with all p-values < 0.05 (Fig. 4c-d). Therefore, we identified SLC2A3 and ASNS as critical ferroptosis genes for JAT inhibition in colorectal cancer.

### High expression of SLC2A3 and ASNS correlates with the prognosis of COAD patients

**Fig. 5:**
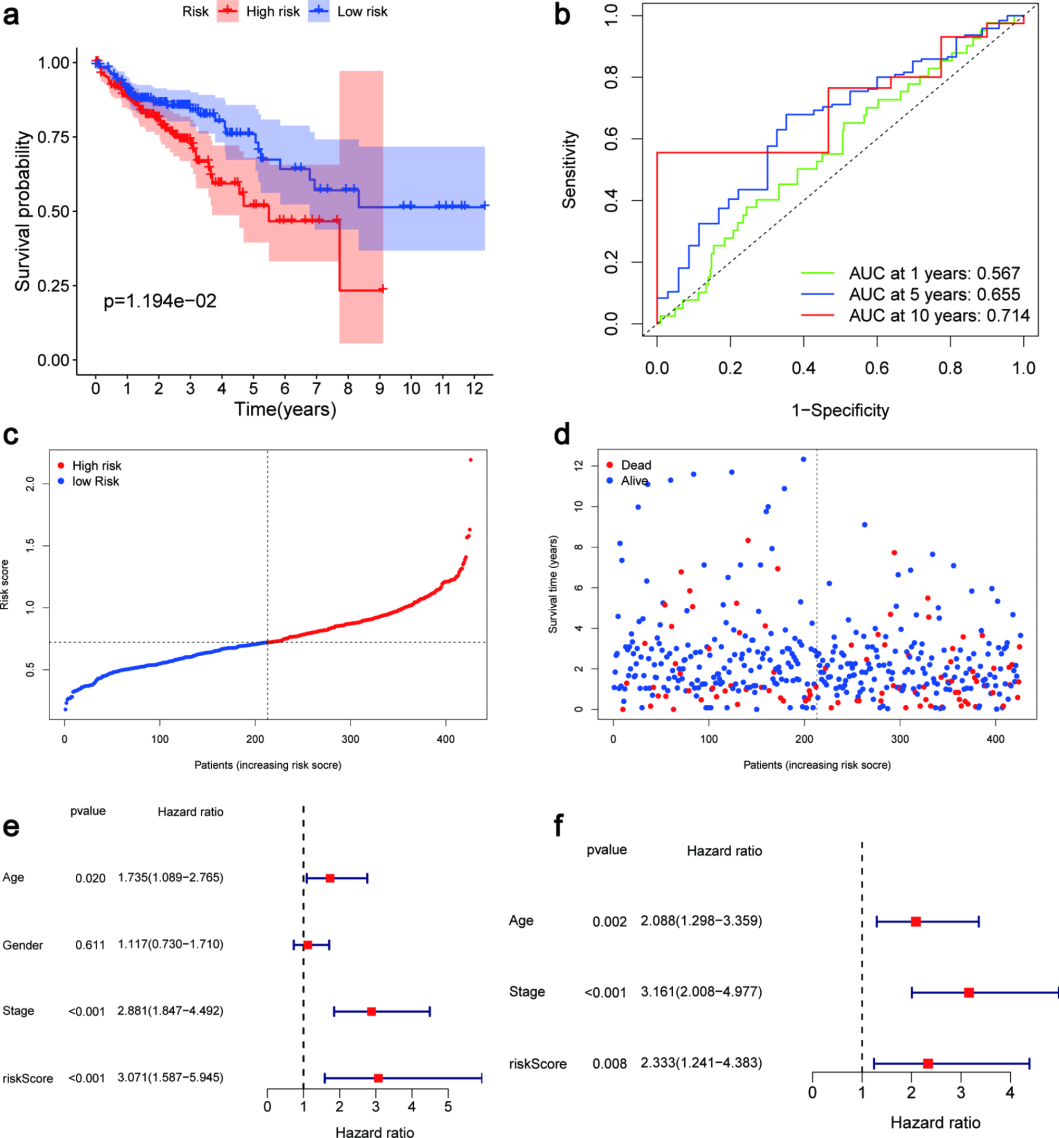
Association between expression levels of critical ferroptosis genes and prognosis of COAD patients. (**a**) The expression of the high-risk and low-risk groups in COAD was verified by survival analysis. (**b**) ROC curve. Validation of the prognostic model, including risk curves (**c**), risk scatter plots (**d**), and independent prognostic analysis (**e**, **f**). P < 0.05 was considered statistically significant

We analyzed gene expression, survival prognosis, and clinical staging of SLC2A3 and ASNS to further explore the core targets of JAT to inhibit COAD growth. We investigated the expression of SLC2A3 and ASNS in tumor and normal groups using the R package, and consistently observed high expression levels of SLC2A3 (p = 0.001) and ASNS (p = 2.708e-15) in the tumor group (Fig. 4e-f). Pairwise differential analysis results showed in Fig. 4g-h (SLC2A3: p = 0.013, ASNS: p = 9.039e-10). Moreover, survival analysis indicated that high expression of SLC2A3 (p = 0.033) was associated with a worse prognosis (Fig. 4i). Figure 4j was the ASNS survival curve (p = 0.03). The potential relationship of SLC2A3 and ASNS with clinical staging was also analyzed (Fig. 4k-l). And using the SLC2A3 and ASNS genes, we calculated risk scores for each patient in the COAD dataset. Based on the median risk score, the 514 patients were divided into high-risk and low-risk groups. Survival analysis revealed that patients in the high-risk group had a significantly worse prognosis compared to those in the low-risk group (p = 1.194e-02) (Fig. 5a). The ROC curves showed that the AUCs at 1, 5, and 10 years were 0.567, 0.655, and 0.714, respectively (Fig. 5b). The risk profile graph illustrated an increasing risk and higher mortality with longer survival time (Fig. 5c-d). Furthermore, we evaluated the independent prognostic value of the prognostic genes by univariate and multivariate Cox regression analysis. The univariate Cox regression results demonstrated that age (p = 0.020), stage (p < 0.001), and risk score (p < 0.001) were risk factors for patient survival (Fig. 5e). The multivariate Cox regression results confirmed that age (p = 0.002), stage (p < 0.001), and risk score (p = 0.008) were independent risk factors for patient survival (Fig. 5f). In conclusion, these results demonstrate that risk scores calculated based on SLC2A3 and ASNS can effectively classify the COAD cohort into high-risk and low-risk groups, enabling the assessment of survival prognosis in COAD patients.

## Discussion

There are many ways to treat colorectal cancer, but most patients experience side effects or recurrence. A large amount of literature shows that the therapeutic results of TCM for the disease have advanced the progress of modern medicine[18]. Inspired by this, we explored the antitumor activity of JAT in colorectal cancer cell lines using the bioactive component JAT extracted from the Chinese herbal medicine *Stephania Epigaea Lo* as experimental material. This study determined the effect levels of JAT on SW480 cells. The cytotoxicity of 12.5, 25, 50, and 75 µM JAT was effective in inhibited the survival of SW480 cells and showed time-dependent and dose-dependent effects. In addition, JAT did not promote SW480 cells apoptosis.

With the development of high-throughput sequencing and bioinformatics, the in-depth study of JAT inhibition of SW480 cells proliferation has been facilitated. In this study, we predicted novel biomarkers of JAT inhibition of colorectal cancer development by high-throughput sequencing. After JAT treatment of SW480 cells, we identified 244 differentially expressed genes using high-throughput sequencing. There have been reports that MAPK, Wnt, and P53 signaling pathway are typical cancer-related pathways that are closely associated with cancer development, metastasis and invasion [19–23]. Multiple studies have indicated that pathways could play a tumor suppressive role in COAD [24–26]. In our study, differentially expressed genes were enrichment in MAPK, Wnt, and P53 signaling pathway. MAPK, Wnt, and P53 signaling pathways are important pathways for JAT in the treatment of COAD. In addition, KEGG and GO enrichment analysis revealed that differentially expressed genes were associated with multiple lipid metabolism, iron ion binding, and ferroptosis. Notably, these characteristics align with the main features of ferroptosis, which involve iron ion accumulation and lipid peroxidation [27]. Based on this evidence, we propose that the inhibition of SW480 cells growth by JAT is associated with ferroptosis.

Ferroptosis contributes to the effectiveness of radiotherapy and chemotherapy for colorectal cancer [28, 29]. We further assessed the ferroptosis gene expression levels of SLC2A3 and ASNS in JAT-treated SW480 cells by RT-qPCR and confirmed that they were significantly highly expressed in COAD. Subsequently, this conclusion was validated by TCGA data that SLC2A3 and ASNS were highly expressed and statistically significant in COAD. Notably, COAD patients with high SLC2A3 expression had a worse prognosis. The literature shows that COAD patients with high SLC2A3 expression have significantly lower OS and may also promote COAD progression through the regulation of EMT and PD-L1 [30–32]. Consistent with these results, our study reveals the role of SLC2A3 in COAD progression and prognosis. 

In conclusion, our study demonstrated the growth-suppressive effects of JAT in SW480 cells. Moreover, through high-throughput sequencing analysis, we found that JAT exerts its inhibitory effects on COAD growth by regulating the expression of ferroptosis-related genes. This offers a promising drug treatment for colorectal cancer.

### Electronic supplementary material

Below is the link to the electronic supplementary material.

Supplementary Material 1

Supplementary Material 2

Supplementary Material 3

Supplementary Material 4

Supplementary Material 5

Supplementary Material 6

## Data Availability

The supplementary material in the article are available at the National Genomics Data Center (NGDC): https://ngdc.cncb.ac.cn/omix/preview/MgRkIMXj.

## Abbreviations

JAT: *Jatrorrhizine*
TCM: Traditional Chinese Medicine
MTT: 3-(4, 5-dimethylthiazol-2-yl)-2, 5-diphenyltetrazolium bromide
TCGA: The Cancer Genome Atlas
COAD: Colorectal cancer
KEGG: Kyoto Encyclopedia of Genes and Genomes;
GO: Gene Ontology
PPI: Protein-protein interaction
SLC2A3: Solute carrier family 2 member 3
DDIT3: DNA damage inducible transcript 3
ASNS: Asparagine synthetase (glutamine-hydrolyzing)
PBS: Phosphate buffered solution
DMSO: Methyl sulfoxide
FerrDb: Ferroptosis database
NCBI: National Center for Biotechnology Information
MAPK: Mitogen-activated protein kinase
ROC: Receiver operating characteristic
AUC: Area under roc curve
EMT: Epithelial-mesenchymal transition
