# Supplementary material for: High-throughput sequencing reveals Jatrorrhizine inhibits colorectal cancer growth by ferroptosis-related genes
Source: BMC Med Genomics. 2023 Sep 14;16:217. doi: 10.1186/s12920-023-01619-3 (PMC10500743; doi:10.1186/s12920-023-01619-3)
Supplement: Supplementary file 6 — Supplementary Material 6 [file 12920_2023_1619_MOESM6_ESM.docx]

**Additional file 1**

Table S1. Primers used in the present study.

Table S2. High-throughput sequencing of 244 differentially expressed genes.

Table S3. KEGG enrichment analysis of differentially expressed genes.

Table S4. A list of the 424 differentially expressed genes associated with COAD in TCGA.

Table S5. A list of the ferroptosis-related 53 differentially expressed genes.
